# Antimicrobial activity of extracts from *Tamarindus indica* L. leaves

**DOI:** 10.4103/0973-1296.66944

**Published:** 2010

**Authors:** Julio César Escalona-Arranz, Renato Péres-Roses, Imilci Urdaneta-Laffita, Miladis Isabel Camacho-Pozo, Jesús Rodríguez-Amado, Irina Licea-Jiménez

**Affiliations:** *Department of Pharmacy, Oriente University, Santiago de Cuba, Cuba*; 1 *Centre for Studies in Industrial Biotechnology, Oriente University, Santiago de Cuba, Cuba*

**Keywords:** Antimicrobial, essential oils, flavonoids, *Tamarindus indica* L, total phenols

## Abstract

*Tamarindus indica* L. leaves are reported worldwide as antibacterial and antifungal agents; however, this observation is not completely accurate in the case of Cuba. In this article, decoctions from fresh and sun dried leaves, as well as fluid extracts prepared with 30 and 70% ethanol-water and the pure essential oil from tamarind leaves were microbiologically tested against *Bacillus subtilis, Enterococcus faecalis, Staphylococcus aureus, Escherichia coli, Salmonella typhimurium, Pseudomona aeruginosa* and *Candida albicans*. Aqueous and fluid extracts were previously characterized by spectrophotometric determination of their total phenols and flavonoids, while the essential oil was chemically evaluated by gas chromatography/mass spectroscopy (GC/MS). Experimental data suggest phenols as active compounds against *B. subtilis* cultures, but not against other microorganisms. On the other hand, the essential oil exhibited a good antimicrobial spectrum when pure, but its relative low concentrations in common folk preparations do not allow for any good activity in these extracts.

## INTRODUCTION

*Tamarindus indica* L. or tamarind, as it is commonly known, is a medium-sized tree belonging to the *Caesalpinaceae* family. Tamarind has been used for centuries as a medicinal plant; its fruits are the most valuable part which have often been reported as curative in several pharmacopoeias. Nevertheless, other plant parts have been less studied. The leaves have a proven hepatoprotective activity associated with the presence of polyhydroxylated compounds, with many of them of a flavonolic nature.[1,2] The seeds and the bark also have medicinal properties.

Due to their antimicrobial, antifungal and antiseptic effects, tamarind leaves have an extensive ethnobotanical use in many areas of Latin America such as Mexico, Puerto Rico, and Trinidad and Tobago, and in other continents like Asia and Africa.[3–6] Within the multi-ethnic Cuban population – very closely related to other Caribbean countries such as Puerto Rico, Trinidad and Tobago and Mexico, and with a great influence of the African and Asian cultures – this pharmacological report is associated only with the traditional use of the plant by African slaves for the treatment of infectious diseases, mainly, intestinal disorders.[7] At present, this use is merely restricted to a few mountainous areas and is not usually reported in local ethnobotanical studies.

It is well known that different climatic, ground and growing conditions can modify qualitatively and quantitatively the chemical composition of the plant and therefore its pharmacological uses. In the specific case of tamarind, a difference in the chemical composition of the fruit pulp essential oil was found between the species that grows in Cuba[8] and the one that grows in Egypt.[9]

In a recent work conducted by our research group on leaves, we reported, for the first time, a total of 13 essential oils in which benzyl benzoate and limonene are the major compounds, followed by hexadecanol and pentadecanol.[10] It is widely accepted that essential oils are one of the plant’s main secondary metabolites involved in antimicrobial and antiseptic activities, in which thyme oil is one of the most significant.[11–13]

Leaves also present good levels of protein, fat, fiber, and some vitamins such as thiamine, riboflavin, niacin, ascorbic acid and β-carotene.[14] Flavonoid and other polyphenols are metabolites that have been also found in tamarind leaves;[15] these compounds have a proven record as antimicrobial agents in many other plants.

In studies conducted in Nigeria, flavonoids and polyphenols in association with alkaloids, were linked to the antimicrobial activity of tamarind leaves.[16] The capacity of flavonoids and polyphenols to be extracted by hot water and into a higher degree by hydroalcoholic solutions gives them an important role in many traditional remedies.

Considering the abovementioned remarks, the purpose of our research is to find a phytochemical and microbiological explanation to the relatively poor use of tamarind leaves in Cuba.

## MATERIALS AND METHODS

### Plant material

Tamarind leaves were collected from a tamarind population in Santiago de Cuba, eastern part of Cuba, and were previously identified by Dr. Jorge Sierra Calzado. A voucher specimen registered as 052216 was deposited in the docent section of BSC herbarium at the biology department of Oriente University.

### Preparation of plant extracts

Decoction was a traditional method selected to prepare the medicinal extracts. We used both fresh and sun dried leaves. Doses employed were 10 g of drug in 100 ml water as described in pharmacopoeias, and a more concentrated extract with a dose of 30 g of drug in 100ml water. In all cases, the volume was made to 100 ml with distilled water at the end of the formulation. Fluid extracts were prepared by the percolation method, using ethanol 30 and 70%. Both the fluid extracts were obtained from 30 g of powdered sun dried leaves and were concentrated by reduced pressure with temperatures below 50°C. For the essential oil, 200 g of fresh leaves was harvested and immediately hydrodistilled for 2 h in a “Clevenger-type apparatus”. Pure *n*-hexane was added to avoid losing the more polar essential oils into the water.

### Phytochemical characterization

For all decoctions and fluid extracts, a spectrophotometric quantification of total phenols and flavonoids was developed. The total phenolic content was estimated using the Folin-Ciocalteau reagent.[17] In brief, the extract was dissolved in water and an aliquot of this solution was added to 2 ml of 2% Na_2_ CO_3_, and after 2 min, 1 ml of Folin reagent was added. After 15 min, the absorbance was measured at 700 nm. The total phenol content was expressed as tannic acid equivalents. The flavonoid content was determined according to the AlCl_3_ method.[18] Briefly, a final volume of extract solution was adjusted to 10 ml with absolute ethanol. Subsequently, 1 ml of 2% AlCl_3_ was added and then the absorbance of sample solution was measured at 430 nm. Data were given as quercetin equivalents. All the measurements were made using a CECIL CE7-200 UV-visible spectrophotometer. A qualitative test for essential oil was also developed for these extracts. Essential oil was characterized by gas chromatography/mass spectrometry (GC/MS) analysis developed on a FISONS Trio 1000 system. An SPB-1 fused silica column of 30 m × 0.32 mm, 0.25 µm film thickness was used, with a temperature program from 70° to 250°C at 4°C/min. Carrier gas (helium) flow rate was 1 ml/min and mass spectra were measured by electron ionization (EI) at 70 eV. Identification was made comparing mass spectra and GC retention indices (RI) with those of our IDENT data bank and with the results of previous works of our group.[10] Some mass spectra were also compared with literature data.[19]

### Test microorganisms

Microorganism strains used were supplied by the Center for Studies in Industrial Biotechnology, Oriente University. *Bacillus subtilis* ATCC 6633, *Enterococcus faecalis* ATCC 29212, *Staphylococcus aureus* ATCC 25923, *Escherichia coli* ATCC 25922, *Salmonella typhimurium* ATCC 14028, *Pseudomona aeruginosa* ATCC 27853 and *Candida albicans* CCEBI 2048 (yeast) were the species used. The bacteria grew on a nutrient Agar Mueller-Hinton (UNI-CHEM) (stationary culture) for 48 h at 37°C, followed by inoculation in nutrient broth (UNI-CHEM). Turbidity was corrected by adding isotonic sodium chloride solution until 10^8^ colony-forming units (CFU/ml) were attained.[20] For yeast, PDA (Sigma, USA) was employed for incubation and inoculation.

### Evaluation of antimicrobial activity

The plate diffusion method in sterile 20 ml petri dishes was used as an antimicrobial test. Inoculated plates were incubated at 37°C for 48 h in the case of *C. albicans* and for 24 h for the rest of the microorganisms. The antibacterial activity of the tested substances was shown by a clear zone of inhibition around the application point. Seven tamarind leaf extracts were evaluated as well as solvents and some antibacterial substances used as references. The dose of decoctions and fluid extracts was 10 µl/plate, and for tamarind essential oil the dose was 4 µl/plate. Positive controls were gentamycin (10 UI), ketoconazole (30 µg) and thyme oil (a BDH pharmacological extract), while solvents were employed as negative control.

### Determination of the minimum inhibitory concentration and minimum bactericide concentration

The broth dilution method approved by the National Committee for Clinical Laboratory Standard (NCCLS) was followed for those extracts that exhibited some activity in the plate diffusion method. Briefly, for all extracts, a series of twofold dilutions was prepared in 1 ml of Mueller-Hinton broth. Aqueous extracts ranged from 0.15 to 0.001 g/ml (leaves weight/volume), while for hydroalcoholic fluid extracts, the doses evaluated varied from 1.5 to 0.01 g/ml (leaves weight/volume). For the essential oils, doses ranged from the equivalent of 40 to 0.31 µl. Test microorganisms were previously diluted to 0.5 McFarland turbidity standard for bacterial isolates. Other tubes containing only nutrient broth and the standard antibiotic gentamycin were also seeded with the test organisms to serve as controls. All the tubes were incubated at 35°C for 24 h, while tubes containing yeast cultures were incubated for 48 h. After incubation, the tubes were examined for microbial growth by observing turbidity. Those visual observations were confirmed by measuring the optical density of the solution at 620 nm in the spectrophotometer aforementioned, establishing the minimum inhibitory concentration (MIC). To determine the minimum bactericide concentration (MBC), aliquots of 100 µl from all dilutions not showing any growth were inoculated on sterile Mueller-Hinton agar plates. Inoculated plates were incubated at 35°C for 24 h for all bacteria, while those inoculated with fungi were incubated for 48 h. MBCs were determined as the lowest concentration in which the extract evaluated did not allow growth of organisms on the agar plate. The presence of one or two colonies was disregarded.

### Statistical analysis

A lineal regression was carried out in order to establish a calibration curve for metabolite quantification. To show the differences between all extracts in total phenol and flavonoid concentrations, an analysis of variance (ANOVA) was performed. Significant differences between means were determined by Duncan’s multiple range tests. *P* values < 0.05 were regarded as significant. Software employed was Statistic version 6.1 for windows.

## RESULTS

### Phytochemical characterization

In aqueous extracts, fresh leaves’ decoctions exhibited higher concentration of phenols but minor levels of flavonoids in comparison with dried leaves’ decoctions which showed lower concentration of phenols but higher of flavonoids. Differences with statistical significances superior to 95% were found between aqueous extracts only with regard to the total phenol content. In the case of total flavonoids, no statistical difference was found between extracts with the same leaves content, irrespective of whether they were fresh or sun dried. For the fluid extracts, differences with statistical significances superior to 95% were found between both the extracts in relation to total phenol and flavonoids. The differences between the 30 and 10% decoctions vary in a range from 2.34 to 1.54 in fresh and sun dried leaves, respectively, for total polyphenols, while for flavonoids they vary from 2.2 to 2.03 [Table 1].

**Table 1:** Total polyphenols and flavonoids average concentration (µg/ml)

	Fresh leaves		Sun dried leaves			
	D. 10% (w/v)	D. 30% (w/v)	D. 10% (w/v)	D. 30% (w/v)	FE. 30% ethanol	FE. 70% ethanol
Polyphenols (µg/ml)	14.84	34.72	9.82	15.14	16.47	18.54
Flavonoids (µg/ml)	0.050	0.110	0.058	0.118	1.087	3.498

D.: decoction, FE.: fluid extract

In all the cases, the fluid extract with ethanol 70% showed the highest concentration for total phenol and flavonoid concentration. In spite of these statistical differences, extracts obtained from sun dried leaves (decoction 30% and fluid extracts) showed concentrations of total phenol in the same order, but in the case of total flavonoids, these differences between the evaluated extracts were remarkably the highest [Table 1]. Results of the characterization of the essential oils from tamarind leaves were similar to those previously obtained by our research group,[10] with a total of 13 essential oils characterized, of which benzyl benzoate is still the main constituent (40.9%), followed by limonene and hexadecanol [Table 2].

**Table 2:** Chemical composition of tamarind leaves’ essential oil

Compound	Pino *et al*,[1] 2002 (%)	Present study
(*E*)-2-hexanal	1.7	1.7
α-Pinene	1.0	0.9
β-Pinene	1.4	1.3
*p*-Cymene	0.6	0.4
Limonene	24.4	24.7
(*E*)-β-Ocimene	t	t
Linalool	1.0	1.1
Linalool anthranilate	4.7	4.8
α-Terpineol	0.7	0.5
Nerol	1.0	1.5
Benzyl benzoate	40.6	40.9
Pentadecanol	8.2	8.3
Hexadecanol	12.4	11.9

t, Trace (≤0.1%)

### Antimicrobial activity

Simple aqueous extracts were active against a few microorganisms. On the other hand, the essential oil of *T. indica* L. and the fluid extracts exhibited a broad spectrum of antimicrobial activity [Table 3]. The determination of MIC and MBC showed *St. aureus* with the highest MIC and MBC with the single exception of 70% ethanol fluid extract, while *B. subtilis* was the most sensitive microorganism to tamarind extracts. All MBCs could be calculated for tamarind essential oil and 70% ethanol fluid extract with the logical exceptions of *P. aeruginosa* and *Sa. typhimurium*, respectively [Table 4].

**Table 3:** Results obtained in the microbiological assay by plate diffusion method

	FL 10%	FL 30%	DL 10%	DL 30%	FE. 30%	FE. 70%	TamEO	ThyEO	Gentam	Ketoco
*St. aureus*	–	+	+	+	+	+	+	+	+	–
*En. faecalis*	–	–	–	–	+	+	+	+	+	–
*B. subtilis*	+	+	+	+	+	+	+	+	+	–
*P. aeruginosa*	–	–	–	–	+	+	–	–	+	–
*Es. coli*	–	–	–	+	+	+	+	+	+	–
*Sa. typhimurium*	–	–	+	+	–	–	+	+	+	–
*C. albicans*	–	–	–	–	–	–	+	+	–	+

FL: fresh leaves’ decoctions, DL: dried leaves’ decoctions, FE.: fluid extract, TamEO: tamarind essential oil, ThyEO: thyme essential oil, Gentam: gentamycin, Ketoco: ketoconazole

**Table 4:** MIC and MBC for tamarind extracts (leaves weight/volume)

Species	FL (g/ml)		DL (g/ml)		FE. 30% (g/ml)		FE. 70% (g/ml)		TamEO (µl)	
	MBC	MIC	MBC	MIC	MBC	MIC	MBC	MIC	MBC	MIC
*St. aureus*	na*	>0.15	>0.15	0.075	1.5	0.047	0.75	0.094	20.0	2.50
*En. faecalis*	—	—	—	—	>1.5	0.094	0.75	0.094	10.0	1.25
*B. subtilis*	0.15	0.019	>0.15	0.038	1.5	0.187	1.5	0.187	10.0	0.62
*P. aeruginosa*	—	—	—	—	1.5	0.187	1.5	0.187	—	—
*Es. coli*	—	—	>0.15	0.075	1.5	0.187	1.5	0.187	10.0	1.25
*Sa. typhimurium*	—	—	>0.15	0.075	—	—	—	1.5	10.0	1.25
*C. albicans*	—	—	—	—	—	—	—	—	40.0	10.0

FL: fresh leaves’ extract, DL: dried leaves’ extract, FE.: fluid extract, TamEO: tamarind essential oil, na* Not applied at the dose evaluated

## DISCUSSION

From the chemical point of view, tamarind leaves are considered as an important source of polyphenols and their derivatives, even when the solvents and methods employed for their extraction are less than ideal, in particular, for the flavonoid type compounds. In the essential oil extract, the presence of nerol and linalool can offer potential antimicrobial activity to the essential oil, but these substances appear in low concentrations. In contrast, the predominant benzyl benzoate is not recognized as a good antimicrobial agent against the bacterial strain used.

The antimicrobial activity of the pure essential oil was higher than those found in the aqueous or hydroalcoholic extracts and was similar to thyme oil (our reference as natural antimicrobial agent). *B. subtilis* was the most sensitive bacteria against any kind of *Tamarindus* extract, whereas *P. aeruginosa* and *C. albicans* remained resistant to a large part of the natural extracts evaluated. In the case of *P. aeruginosa*, this resistance was found even with the natural reference substance, thyme oil. Nevertheless, all the microorganisms tested were sensitive to a t least to one of the extracts evaluated, denoting a broad spectrum of action of tamarind leaves’ metabolites.

In a general sense, aqueous and hydroalcoholic extracts are more effective against gram positive than gram negative bacteria. This behavior was observed by Meléndez 2006 for tamarind leaves too; nevertheless, a different trend has been observed for *E. coli*, against which fluid extracts are slightly effective, but their inhibition ratios are too far from those reported in the very similar Puerto Rico flora.[4] On the other hand, tamarind essential oil exhibits an acceptable inhibition diameter against *Es. coli* and this behavior coincides with the activity of limonene,[21] one of the major constituents of the tamarind leaves’ essence. Other essential oils present in tamarind leaves such as α and β-pinene, linalool and nerol have proven activity against *Es. coli* and other bacteria, but they are predominantly inactive against *P. aeruginosa* as it occurs with tamarind leaves’ essence.

With regard to the diverse nature of the solvents employed in this research, it is evident that a diminution of the polarity helps to extract the bioactive metabolites. On the other hand, to obtain a broad spectrum extract from tamarind leaves, it is necessary to develop extraction methods with a certain level of complexity (fluid extracts or essential oil isolation), which are not common within the average population.

### Correlation between chemical composition and the antimicrobial activity

Only for *B. subtilis*, all MICs and almost all MBCs were calculated in the extracts characterized by phenol and flavonoid levels, allowing for a correlation. To correlate the influence of total phenols and flavonoids over this bacterium, it is necessary to estimate the real concentration in which these metabolites are present in the MIC and MBC previously calculated. As all evaluated concentrations were from a series of twofold dilution of a more concentrated and well-characterized solution, a single mathematical operation gives an opportunity to estimate the levels in which these substances are present. Against this bacterium, total phenol levels at the MIC in fresh and sun dried leaves’ decoction extracts were 2.788 and 3.666 mg/ml, respectively, and for 30 and 70% of ethanol fluid extracts, the levels were 3.088 and 3.458 mg/ml, respectively. In the case of MBC, values of 22.246, 24.774 and 27.741 mg/ml were obtained for decoction with fresh leaves, 30 and 70% ethanol fluid extracts, respectively. As a similar concentration of total phenols was found in the calculated MIC and MBC, it suggests, at least some role for these compounds in the antimicrobial activity against this bacterium. Similarly, flavonoid levels in MIC were 0.009 and 0.022 mg/ml for decoctions in fresh and sun dried leaves, respectively, whereas for 30 and 70% ethanol fluid extracts, the levels were 0.207 and 0.690 mg/ml, respectively. The MBC levels of fresh leaves’ decoction and 30 and 70% ethanol fluid extracts were 0.074, 1.658 and 5.537 mg/ml, respectively. These dissimilar values suggest a poor influence of flavonoids in the antimicrobial activity against this bacterium.

In those bacteria in which three positive assays were reported (*St. aureus and Es. coli*), dissimilar values of phenol and flavonoid concentration were obtained, suggesting a low influence of these compounds on the activity. This kind of analysis was limited to only these bacteria, because others showed only two or less positive assays, nevertheless, in *P. aeruginosa* the values of phenol concentration in MIC and MBC were very close in the two positive extracts (fluid extracts in ethanol at 30 and 70%), indicating a possible relationship with the activity.

In spite of these results, the most valuable antimicrobial activity of any plant extract is related to its microbiological spectrum. The fact that in fresh leaves’ decoction the amount of polyphenols is comparatively higher than in sun dried leaves’ decoction and is more or less of the same level in the fluid extracts, but with a worse antimicrobial spectrum, looks like these kind of compounds are not the driving force behind the antimicrobial spectrum of tamarind extracts. On the other hand, similar flavonoid concentrations in decoction extracts do not expose a clear relevance in the different spectra observed between sun dried and fresh tamarind leaves’ decoctions, as well as the similar microbiological spectra of the both fluid extracts, while they have different flavonoid concentrations.

Essential oils from tamarind leaves revealed a broad antimicrobial spectrum, but the small quantities in which it has been determined in our tamarind crops, were not enough to show any important activity. Thus, the low antimicrobial activity in fresh leaves decoction extracts could be related to the very low concentration of these compounds in the preparation, and as mentioned before, decoction is the most popular way to prepare traditional medicinal beverages.

In our experimental conditions, we cannot declare that either flavonoid or polyphenols are the compounds responsible for tamarind antimicrobial spectrum, as has been suggested previously by Doughari in 2006.[16] On the other hand, tamarind essential oils look like good antimicrobial agents, but their low concentration in the Cuban climatic conditions do not favour their presence in common water preparations.

## CONCLUSIONS

Tamarind leaves are traditionally used worldwide as an antimicrobial agent. This application is not well accepted by the Cuban population even when multiple varieties of metabolites with a recognized antimicrobial activity have been reported before.

In the light of our results, we can suggest that some compounds found in the essential oils from leaves of the *T. indica* L. that grows in Cuba could be responsible for its antimicrobial activity. Anyway, in our climatic conditions, the poor yield of essential oil production, and in addition, the low levels of the most prominent antimicrobial compounds detected in the tamarind leaves’ essence give us a possible explanation to the relatively poor ethnobotanical reports in Cuba. Nevertheless, we cannot reject the fact that other compounds such as phenols could show some activity against particular kinds of microorganisms. Also, a combination of some of the metabolites found with others types of compounds extracted by other popular remedy preparations could form a natural mixture that reaches the final antimicrobial spectrum by some kind of synergy, as it could have happened with the hydroalcoholic extracts.
